# An Atypical Case of Miller Fisher Syndrome: Classic Clinical Triad Despite Negative Anti-GQ1b Antibody

**DOI:** 10.7759/cureus.80529

**Published:** 2025-03-13

**Authors:** Sami Refai, Ammar Mattar, Neel Patel, Stephen D Hull, Richard Virgilio

**Affiliations:** 1 Internal Medicine, Edward Via College of Osteopathic Medicine, Auburn, USA; 2 Internal Medicine, Grandview Medical Center, Birmingham, USA; 3 Clinical Affairs, Edward Via College of Osteopathic Medicine, Auburn, USA

**Keywords:** gbs variant, guillain barre syndrome (gbs), internal medicine and rheumatology, miller fisher syndrome (mfs), miller fisher variant

## Abstract

Miller Fisher syndrome (MFS), a variant of Guillain-Barré syndrome (GBS), was diagnosed in a 30-year-old patient following a mononucleosis infection. The progression of this patient’s disease began with the classic triad of symptoms (areflexia, ophthalmoplegia, and ataxia) beginning at the head and progressing downward. Most notably, the diaphragm was spared. Ophthalmoplegia, ataxia, and areflexia were among the most defining of symptoms; however, this patient also presented with restlessness in his extremities and urinary hesitancy. Diagnostic workup of this patient included testing for anti-GQ1B antibodies in addition to a thorough history and physical exam. Initial treatment with intravenous immunoglobulin (IVIG) was unsuccessful. Plasmapheresis was ultimately initiated; however, the patient failed to complete treatment due to iatrogenic complications. The patient failed to return to follow-up appointments to determine recovery status. This case underscores the critical importance of a thorough and systematic diagnostic approach in managing atypical presentations of GBS variants.

## Introduction

Guillain-Barré syndrome (GBS) has five unique variants that present in their own respective ways. Although each variant is unique, there is a degree of shared etiology, pathophysiology, and clinical symptomatology that ties each of them together under the umbrella of GBS. GBS is an acute immune-mediating attack on the nerve sheath of the peripheral nervous system. 

Pathophysiologists have determined that autoantibodies develop via molecular mimicry [1], a process involving cross-reactivity of pathogen-borne antigens with native proteins in the body, such as gangliosides or unknown antigens of Schwann cells. This leads to axonal degeneration of nerves in the peripheral nervous system (PNS) and, ultimately, clinical signs and symptoms that resemble demyelinating diseases such as multiple sclerosis. Classic symptoms include paresthesias [2], hyporeflexia [3], cardiac arrhythmias, blood pressure fluctuations [2], respiratory involvement, and ultimately, cranial nerve disruptions. None of these symptoms are permanent, and treatment results in complete remission of symptoms. Relapses have been documented as long as 12 years after the initial insult [4].

According to the common understanding of GBS, five variants of this disease have been discovered among practitioners and researchers, each carrying varying degrees of overlap with each other and respective differences from a diagnostic perspective. Shared diagnostic overlaps include, but are not limited to, areflexia, paresthesias, albuminocytological dissociation in CSF, and the presence of autoantibodies. Acute inflammatory demyelinating polyneuropathy (AIDP) is the most common subtype of GBS and is responsible for up to 90% of cases in the US, Canada, and Europe [2]. AIDP is most commonly associated with *Campylobacter jejuni* and cytomegalovirus infections. Nerve conduction studies typically show signs of demyelination that affect motor neurons before sensory neurons [5]. The progression of AIDP is subacute compared to its rapid counterpart, acute motor and sensory axonal neuropathy (AMSAN). AIDP presents similarly, with the key differentiation being the rapidly progressive course of the disease. A high degree of clinical suspicion is required for AMSAN due to its speed of progression, leading to respiratory failure within days of onset [5].

Bickerstaff and Miller Fisher variants comprise a category of GBS known as the anti-GQ1B antibody syndromes [6]. Clinical suspicion for these variants of GBS requires relatively strict criteria of progressive, relatively symmetrical external ophthalmoplegia and ataxia by four weeks and disturbance of consciousness or hyperreflexia [6]. Bickerstaff brainstem encephalitis (BBE) is a relatively uncommon variant that presents similarly to Miller Fisher syndrome (MFS), except with unique inflammatory brainstem changes [6]. The appearance of altered consciousness versus clear consciousness is also a diagnostic criterion differentiating BBE from MFS [6]. Interestingly, compared to other variants of GBS that traditionally show an ascending pattern of progression, the anti-GQ1B syndromes display a descending pattern that typically begins with ophthalmoplegia and progresses caudally. MFS is among these variants whose clinical progression is a reversal of traditional GBS. Rather than ascending paralysis, as is most commonly described with GBS, MFS begins its nervous system attack on the eyes and progresses caudally [1].

## Case presentation

A 30-year-old male with a past medical history of anxiety, hypertension, and chronic alcohol use presented to the emergency department (ED) with new-onset bilateral extremity weakness. His regular medications included citalopram, lisinopril, omeprazole, chlordiazepoxide, and thiamine. The patient reported consuming approximately half a pint of hard liquor daily. He returned from vacation with a suspected viral illness and was diagnosed with infectious mononucleosis one week after the onset of a sore throat following an urgent care visit.

One month following this viral illness, the patient presented to the ED with complaints of progressive bilateral upper and lower extremity weakness and unsteadiness lasting one to two weeks. He described feeling "wobbly", experiencing multiple falls and frequently running into objects, though still able to ambulate independently. Of note, the patient reported temperature dysregulation, blurred vision and irregular blinking preceding the onset of extremity weakness. He also endorsed genitourinary symptoms, including urinary hesitancy and erectile dysfunction. Neurology was consulted by the ED who suggested simple GBS or MFS. The neurologist concluded it could be MFS due to the classic triad of ataxia, ophthalmoplegia, and areflexia in a descending fashion. The patient was admitted to the medical floor of the hospital due to the clinical progression weakness with a possible threat of respiratory failure. Respirations and oxygen were normal at the time of admission.

The patient was anxious, talkative, and restless. No motor strength deficits were observed, but repetitive blinking and signs suggestive of restless leg syndrome were noted. Reflexes were diminished at 1+, which was deemed borderline. Pulmonary examination was normal. Cardiovascular examination revealed sinus tachycardia on electrocardiogram (EKG). The remainder of the physical exam was unremarkable.

Laboratory studies were performed during intake in the emergency department and at various points during admission, including complete blood count and metabolic panel, and were within normal limits. Imaging, including CXR, CT head, CT lumbar spine, MRI of the brain, and MRI of the lumbar spine, was unremarkable. A lumbar puncture was performed, revealing cerebrospinal fluid (CSF) analysis with albuminocytologic dissociation and an elevated protein level of 55 mg/dL (normal: <40mg/dL). Serology demonstrated elevated Epstein-Barr virus (EBV) immunoglobulin G (IgG) antibodies, consistent with a prior EBV infection. Antibody testing for anti-GQ1B was negative.

Due to the clinical progression of descending symptoms, a diagnosis of MFS of GBS was made despite a negative anti-GQ1B antibody. The patient's clinical presentation of ataxia, blurred vision, irregular blinking, bilateral extremity weakness, and genitourinary symptoms, coupled with CSF findings of albuminocytologic dissociation, was consistent with a diagnosis of Guillain-Barré syndrome (GBS). The absence of anti-GQ1B antibodies could suggest a diagnosis of the classic form of GBS rather than the MFS variant, despite the descending paralysis pattern. The unusual progression of symptoms, including early ocular involvement, raises the possibility of an atypical presentation of GBS.

Treatment was initiated after the diagnosis of GBS was confirmed. The patient, with a newly suggested MFS variant of GBS, began first-line treatment with IVIG. His symptoms consisted of ataxia, urinary hesitancy, erectile dysfunction, blurry vision, shortness of breath, upper extremity weakness, tachycardia, and temperature dysregulation. He was initially treated on the medical floor of the hospital with five days of IVIG with temporary improvement and was discharged home. Seven days after discharge, the patient returned to the ED and was readmitted for recurrence of symptoms, including tachycardia and hypertension. The patient also admitted to consuming 16oz of hard alcohol during the time between admissions. After treatment failure, plasma exchange (PLEX) was recommended for five sessions over a total of 10 days.

**Figure 1:**
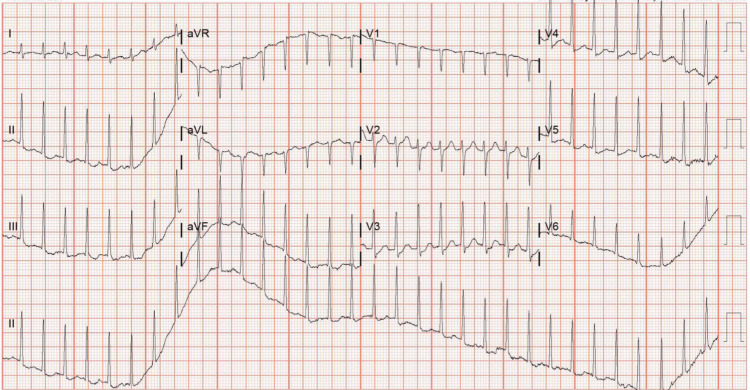
Patient's normal complex tachycardia resulting an emergency response team and cessation of plasma exchange.

The initial session of PLEX had no complications aside from nausea and vomiting. The patient reported no relief from his symptoms. After completing the second round of PLEX on the fourth day, the patient began experiencing extreme lightheadedness. Further cardiac evaluation showed the patient to have a narrow complex tachycardia at 220 bpm, as seen in Figure 1.

The emergency response team stabilized the patient with diltiazem (Code Blue) and transferred him to the ICU for monitoring. After this incident, PLEX was discontinued under the recommendation of the patient's hospitalist, due to the concern of reemergent arrhythmias. Shortly after the PLEX treatment failure, the hospitalist discharged the patient from the ICU at the patient’s request. The decision to discharge was made at the discretion of the hospitalist; the patient did not leave against medical advice.

Two weeks after discharge from his hospital readmission, the patient followed up with the neurology department, which concluded that the patient did not require further PLEX treatment. Across multiple reassessments, the patient stopped vomiting, no longer had difficulty urinating, showed improved reflexes, and maintained sobriety since discharge. During this neurology visit, an alcohol cessation program was discussed and agreed upon by the patient. A nerve conduction study was also discussed for the next appointment; however, the patient failed to follow up. The patient's recovery status is unknown.

## Discussion

MFS most commonly presents with a triad of ataxia, ophthalmoplegia, and areflexia that subsequently appear after a bacterial or viral infection [7]. Other less common symptoms include facial paralysis, pupillary palsies, dysesthesia in the extremities, blepharoptosis, and mild muscular weakness [7]. This form of GBS can be caused by infectious agents such as *Mycoplasma pneumoniae*, cytomegalovirus, Epstein-Barr virus, and human immunodeficiency virus, although the two microbes that most commonly cause GBS are *Haemophilus influenzae *and *Campylobacter jejuni* [7,8]. The diagnosis of MFS is made by conducting a complete history and physical along with unremarkable findings on MRI and CT, the presence of Anti-GQ1b antibodies in the serum, and albuminocytologic dissociation [7]. It should also be noted that only 50% of patients have albuminocytologic dissociation on initial analysis; therefore, if it is not present, GBS and MFS cannot be ruled out [8].

A notable aspect of our patient’s case was the lack of anti-GQ1b antibodies. While these antibodies are present in up to 95% of patients during the acute phase of MFS and are highly specific and sensitive for confirming the diagnosis, a negative result does not rule out MFS [9].

Clinicians should be aware that MFS has overlapping symptomology with many other pathologies. Descending paralysis and ptosis can also manifest in botulism [10]. Areflexia, which is part of the triad presentation of MFS, can also be seen in cobalamin deficiencies or other neurological pathologies such as amyotrophic lateral sclerosis (ALS), polio, or tabes dorsalis [7]. MFS is most likely caused by molecular mimicry between the infectious agent’s antigen and the peripheral nerves [8]. The weakness of the muscles around the eye is most likely caused by IgG anti-GQ1b antibodies that affect the neuromuscular junction between the cranial nerves and the muscles of the eyes [8].

Wernicke’s encephalopathy is a pathology with overlapping clinical features such as altered mental status, ophthalmic symptoms, and ataxia [11]. Thiamine deficiency is usually the precipitating factor and alcohol use disorder is a risk factor for this condition [11]. However, this individual’s thiamine levels were within normal limits, therefore ruling out Wernicke’s encephalopathy. Gait disturbances and motor strength weakness can also be manifestations of vitamin B12 deficiency [12]. Cobalamin deficiency can lead to demyelination of the dorsal and lateral columns of the spinal cord [12]. This nutritional deficiency was also ruled out because his B12 levels were also within normal limits [12].

Chronic alcoholism also plays a role in the progression and recovery of GBS. According to a study by Pagaling et al., GBS patients with chronic alcoholism have decreased odds of developing common symptoms of GBS such as tachycardia, blood pressure fluctuations, and other autonomic disturbances [13]. The protective nature of chronic alcoholism on autonomic disturbances is not fully understood [13]. In contrast, chronic alcoholism can negatively impact the efficacy of IVIG and PLEX in GBS patients [14]. A retrospective observational study stratified patients based on various risk factors such as alcoholism revealed that those with this risk factor (OR=5.148; CI=1.234-21.472, p=0.025), were at risk of no improvement despite treatment [15].

The mainstay treatment for MFS primarily focuses on symptomatic treatment and respiratory support if needed [8]. IVIG or plasmapheresis can be considered if a patient has a severe form of this pathology [8]. No evidence currently shows which of these two types of treatment will lead to lower mortality, length of intubation, or disability [8]. However, checking for IgA deficiency is pertinent if the patient receives IVIG, due to the risk of anaphylaxis if they are IgA deficient. Corticosteroids are not recommended in the treatment of MFS unless the patient is experiencing neuropathic pain [8]. Due to the multifaceted pain profile accompanying MFS, a combination of medications such as gabapentin, pregabalin, or amitriptyline can be used [8]. If an opioid is deemed necessary to aid in pain control, it should be used with caution as this medication class further suppresses respiratory drive [8]. Since MFS can cause urinary retention and dysphagia, urinary catheters and nasogastric feeding may be warranted [8].

## Conclusions

This case illustrates an atypical presentation of MFS in a 30-year-old male following infectious mononucleosis, with descending paralysis and early ocular involvement. Despite the absence of anti-GQ1b antibodies, clinical features such as ophthalmoplegia, ataxia, areflexia, and albuminocytologic dissociation in the cerebrospinal fluid supported the diagnosis. While up to 95% of MFS cases present with anti-GQ1b antibodies, this case demonstrates that a negative result does not exclude the diagnosis. Nutritional deficiency that can be caused by alcohol use disorders, such as Wernicke’s encephalopathy and subacute combined degeneration, were ruled out since his cobalamin and thiamine levels were within normal limits. Early recognition and diagnosis are critical in guiding treatment and improving outcomes, even in atypical presentations. This case underscores the importance of considering MFS in patients with descending neurological deficits, particularly in the context of prior viral illness, and highlights the need for careful differential diagnosis when anti-GQ1b antibodies are negative.
